# Catering to Inclusion and Diversity With Universal Design for Learning in Asynchronous Online Education: A Self-Determination Theory Perspective

**DOI:** 10.3389/fpsyg.2022.819884

**Published:** 2022-02-21

**Authors:** Murod Ismailov, Thomas K. F. Chiu

**Affiliations:** ^1^Faculty of Humanities and Social Sciences, Center for Education of Global Communication, University of Tsukuba, Tsukuba, Japan; ^2^Department of Curriculum and Instruction, The Chinese University of Hong Kong, Shatin, Hong Kong SAR, China

**Keywords:** universal design for learning, self-determination theory, asynchronous online courses, inclusion, diversity, higher education, mixed methods

## Abstract

The Universal Design for Learning (UDL) guidelines were extensively studied to understand inclusive learning and teaching in higher education. However, to date, there have been few studies that approached UDL-based asynchronous university courses from the needs satisfaction perspective in self-determination theory (SDT). To address this gap, researchers designed and implemented two 15-week asynchronous online courses based on UDL. They then tested their effectiveness with college freshmen (*N* = 225) by adopting a sequential explanatory mixed method. The study aimed to examine (i) whether asynchronous instruction based on UDL catered to inclusion and diversity across gender and academic background and (ii) whether the instructional design supported learner engagement and needs satisfaction. The findings showed that both male and female students as well as Arts and Sciences students equally engaged in the courses and perceived the needs support from the course design. However, the study also found that although universal design supported autonomy and competence, it nonetheless failed to fully satisfy learners’ relatedness needs. The researchers concluded by discussing empirical and theoretical implications.

## Introduction

The growth of inclusive and diverse education in both conventional and online settings trigger the adoption of universal access frameworks that were originally created for architects, engineers, and designers (The Center for Universal Design, 1997; Story et al., 1998; Schreiber, 2017). The universal design for learning (UDL) is built on seven principles of universal design: (1) equitable use, (2) flexibility in use, (3) simple and intuitive, (4) perceptible information, (5) tolerance for error, (6) low physical effort, and (7) size and space for approach and use. These principles lay the foundation for universal design to stress the need for “teaching and learning products and environments to be usable by all people, to the greatest extent possible, without the need for adaptation or specialized design” (Burgstahler, 2021). Crucially, UDL goes beyond accessible design for people with disabilities to make all aspects of the educational experience more inclusive for all stakeholders regardless of gender, race and ethnicity, age, disability, and learning style (Burgstahler, 2015).

Given the growing need for online learning in the post-pandemic environment, it is important to adopt pedagogical designs for inclusion and diversity and examine their impact on online learners’ needs satisfaction, engagement, and performance across demographic and academic characteristics. Such studies are especially needed in regions that traditionally experience issues with inclusion and equity in education (Hatano, 2021). For example, recently, some universities in Japan found that their online learning curricula were inadequately designed to address the needs of all students (Ismailov and Ono, 2021). Due to a limited number of published studies in the region (McEown and Oga-Baldwin, 2019; Ismailov and Ono, 2021; Rivers et al., 2021), this study contributes to the field by providing more evidence on how a UDL-designed course could address inclusion and diversity issues in online learning curricula.

Universal instructional design plays a key role and is used as an umbrella term to describe a pedagogical framework for eliminating barriers to learning and responding to the needs of all learners when designing and delivering courses (Burgstahler, 2021). The design is proactive and benefits all learners regardless of their characteristics, in contrast to providing accommodations for specific learners (Burgstahler, 2015, 2021). For example, Differentiated Instruction (DI) is a pedagogical design for addressing inclusion and diversity. Teachers adapt their teaching, assessment, and grouping strategies to cater to students with diverse learning readiness, profiles, and interests (Tomlinson, 2014). Unlike DI, UDL provides detailed checkpoints for designing curricula that enable all learners to actively engage, feel included, and learn enthusiastically with peers in both online and physical classrooms (Griful-Freixenet et al., 2021). It also supports research on inclusive and diverse instruction by providing more evidence-based course design with measurable tools (Rose and Meyer, 2002; Capp, 2017; Schreiber, 2017).

Online learner differences (other than physiological) can be broadly classified into demographic, academic, cognitive, affective, self-regulation, and motivational characteristics (Martin et al., 2020). While the last four receive much attention in the literature (e.g., Thomas, 2016; Gale et al., 2017; Moriña, 2020), the first two need to be further investigated. For example, previous studies stress the importance of gender inclusion in online courses (Bayeck et al., 2016), in part due to both an increasing and decreasing share of female online learners in some countries and across certain environments, such as massive open online courses or MOOCs (Secreto, 2013; de Souza and Perry, 2021). There are still remaining issues with equity. One study shows that female students may be more active than males (i.e., females tend to view as well as write more and longer posts) but receive the same grade as male students (Lowes et al., 2016). In the instructional design, female students’ efforts and engagement may not result in better academic outcomes. These findings occur in students with different academic achievements (Bradford and Wyatt, 2010). However, several studies find no significant differences between genders in online learning outcomes (Yu and Yu, 2021), as well as students’ engagement and performance (Krasodomska and Godawska, 2020), while other studies suggest that gender and academic stance could predict online learning self-efficacy (Shen et al., 2013). In sum, we are not clear how instructional designs affect students of different genders and academic achievements. Accordingly, the present study examines the degree to which asynchronous courses based on UDL could (i) cater to inclusion and diversity and (ii) affect satisfaction, performance, and engagement. The results of the study can inform teachers and curriculum professionals about the effectiveness of universal design in constructing inclusive asynchronous online courses.

## Literature Review

### Inclusive Pedagogy in a University Context

The literature related to inclusive pedagogy focuses strongly on the inclusion of students with disabilities (Collins et al., 2018; Carballo et al., 2019; Leijen et al., 2021). Teaching practices seem to accommodate the needs of exceptional students, which may not be fully inclusive unless other classroom diversities such as gender and academic background are addressed (Burgstahler, 2021). More studies include parameters other than disability, such as age, gender, ethnicity, academic background, and learning styles in inclusive education (Waitoller and Thorius, 2016; Gale et al., 2017; Grier-Reed and Williams-Wengerd, 2018). Recognizing that everyone could learn better under the right conditions (Moriña, 2020), inclusion in the pedagogical setting is described as a process in which educators *“respect and respond to human differences in ways that include learners in, rather than exclude them from, the daily life of the classroom”* (Florian and Black-Hawkins, 2011, p. 814).

Scholars identify three broad domains with which to approach inclusive pedagogy when teaching students with and without disability: (i) a *belief* that all students bring something of value to the learning environment; (ii) a *design* that values differences while also providing access to and enabling engagement with dominance; and (iii) *actions* that work with students and their communities (Gale et al., 2017; Moriña, 2020). In other words, inclusive pedagogy focuses not only on universally effective ways to transfer and assess content knowledge but also considers a wide range of cognitive, affective and behavioral issues, such as incorrect assumptions about students’ knowledge and skills, differential expectations of students, and quality of relations and respect between teachers and students from different backgrounds (Singh, 2011). Research has also found that students experience a range of academic challenges related to previous educational experiences and academic preparedness, such as language issues contributing to poorer academic and communication skills (Bisai and Singh, 2020), inadequate student support mechanisms, and a lack of understanding of what is expected academically (Stevenson, 2012). Together, this triad of beliefs, designs and actions indicates that a course design must be planned right from the start to be as inclusive and accessible as possible and to satisfy the educational needs of all students (Moriña, 2020).

Regarding the inclusiveness of classroom practices, studies show that effective interventions are associated with a set of common characteristics, such as catering to diverse needs and expectations, proactive and flexible sessions with relevant content and well-timed instruction, collaborative and facilitated environments followed by quality feedback, and using accessible technology and media (Thomas, 2016; Chiu and Hew, 2018; Carballo et al., 2019; Moriña, 2020; Zhao et al., 2021). As opposed to traditional teacher-centered instruction that prioritizes knowledge transmission through monologic lectures with limited interaction and is evaluated based on students’ ability to correctly reproduce such knowledge, inclusive pedagogy employs a constructivist approach to create an inclusive environment for all learners. In other words, teachers approach the classroom as co-creators of knowledge working alongside students rather than in front of them (Grier-Reed and Williams-Wengerd, 2018).

### Universal Design for Online Learner Engagement

**FIGURE 1:**
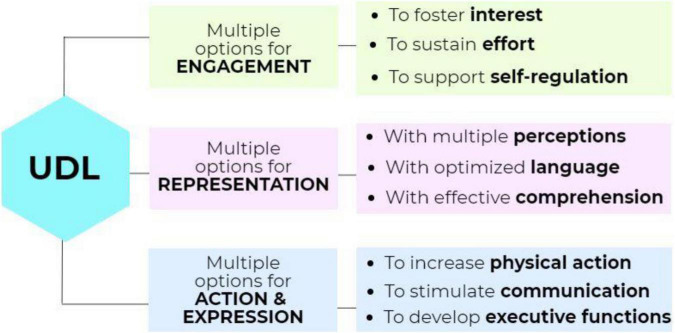
UDL Framework (adapted from CAST, 2018).

Universe design principles are applied in various educational products (websites, software, textbooks) and physical environments (dormitories, classrooms, libraries, student services). The Center for Applied Special Technology (CAST) developed the UDL, especially for designing technology-mediated instruction (Burgstahler, 2015). UDL is *“a research-based set of principles that together form a practical framework for using technology to maximize learning opportunities for every student”* (Rose and Meyer, 2002: preface). The framework recommends presenting course content in multiple ways, providing students with various options for engagement, and facilitating their choices to demonstrate acquired knowledge and skills (Rao and Meo, 2016; Chiu, 2021a,b,c). In addition, drawing from research in neuroscience, the framework helps teachers (i) set appropriate goals for every student, (ii) choose the teaching methods and materials that give every student optimal instructional support, and (iii) ensure the fair and accurate assessment of every student’s progress (Rose and Meyer, 2002; Chiu, 2021a,b,c) by offering multiple options for classroom engagement, representation, and action and expression (see Figure 1).

For example, providing students with multiple options for engagement implies (i) fostering learners’ interest with learning activities that offer individual choice, autonomy, relevance, minimum distractions; (ii) sustaining learners’ effort with tasks that provide clear goals, intellectual challenge, peer collaboration, and timely feedback; (iii) supporting self-learners’ regulation with activities that improve motivation and coping skills as well as self-assessment and reflection.

Second, giving learners more options for representation entails using different tools and media (iv) to support their multiple perceptions through customizable and alternative audio-visual information; (v) optimizing language and symbols using clarified vocabulary, structure, text decoding, illustrations with multiple media; (vi) optimizing comprehension by using background knowledge; patterns, big ideas, visualization, and generalization.

Lastly, the UDL environment also provides many alternatives for action and expression, especially for (vii) increasing physical action using varied response and navigation, optimized access and assistive technologies; (viii) stimulating communication through multiple media and support for practice and performance; (ix) developing executive functions such as goal-setting, planning, strategizing, managing information and monitoring progress (CAST, 2018). Digital technology is a key tool in applying such inclusive pedagogical practices (Schreiber, 2017). Studies identify optimal usability designs, behaviors, and tools to highlight the role of web-based curricula and embedded support when implementing UDL in a classroom setting (Edyburn, 2020). However, the role of digital technology in UDL has been a topic of debate for some time. Some authors suggest that because it provides teachers/students a means of representing/demonstrating knowledge in multiple ways by engaging many students and accommodating diverse needs, technology is a key element of UDL (Spencer, 2011; Capp, 2017). Others argue that learner-centered pedagogy is more vital to the acceptance of UDL (Courey et al., 2012). However, many teachers and authors maintain that integrating technology with sound instructional strategies and curriculum helps create customized and scaffolded learning experiences for students with diverse needs (Capp, 2017; Edyburn, 2020; Burgstahler, 2021; Ismailov and Ono, 2021).

Overall, most studies support the claim made by CAST about the effectiveness of UDL in catering to classroom diversity and increasing access to learning by reducing physical, cognitive, intellectual, and organizational barriers (Rose and Meyer, 2002; Capp, 2017). With some exceptions (e.g., King-Sears et al., 2014), the UDL is found to be effective in improving the learning process across elementary to postsecondary education as well as different subject areas involving students with and without disabilities (Schreiber, 2017).

### Behavioral Engagement in Universal Design for Learning-Based Online Learning

Recent studies focus on how UDL affects learners’ behavioral engagement in online and blended environments (Sanchez-Gordon and Luján-Mora, 2016; Al-Azawei et al., 2017; Rogers-Shaw et al., 2017; Herrara Nieves et al., 2019). They find that using UDL-embedded educational technologies enhance learner willingness and motivation to accept online learning (Al-Azawei et al., 2017), resulting in greater behavioral engagement of all the students. The studies suggest various UDL-based instructional designs. For example, incorporating various assignment formats and flexible options in highly structured formats show that students responded positively to the course design and instructor interaction (Rao et al., 2015; Chiu, 2021a,b,c). Different activities, such as self-evaluation, forums, tests, and tasks, should be provided to students to freely choose the activities they prefer (Fidalgo and Thormann, 2017). Offering students a wide range of stimulating activities, such as tag clouds, mind and concept maps, comment writing, homemade audio-visual, and pictorial tasks, enhance the participation of all the students (Herrara Nieves et al., 2019). Overall, these studies showed that UDL-based designs are more likely to engage all students in online environments, addressing the issues of inclusion and diversity.

### Emotional Engagement in Universal Design for Learning-Based Online Learning

An important emotional aspect of UDL is associated with the instructor’s presence. Studies suggest that the instructor’s nurturing nature and behavior influence how students perceive their needs support (Hinshaw and Gumus, 2013; Stein, 2014; Moriña, 2020). Attentive, approachable, and encouraging teachers are more likely to emotionally engage students in learning in response to their queries (Zhao et al., 2021). They make reasonable adjustments to meet students’ individual needs, provide clear course requirements and expectations, and respond to student queries in a timely manner. Teachers can use UDL approaches to prioritize contextualized, personalized, and culturally relevant content instruction to elevate students’ effort, motivation, and self-regulation (Herrara Nieves et al., 2019).

Moreover, using UDL-based digital technology in online learning can emotionally engage students in online learning by promoting learners’ self-expression and a sense of belonging to a community and fostering learners’ ability to demonstrate what they learn in the virtual classroom (Herrara Nieves et al., 2019). In this learning environment, students express greater emotional engagement due to improved student-student communication and more fun learning (Chiu, 2021a,b,c). However, the research also points out that the affective response may vary depending on the course and its perceived utility and on whether the courses are blended with traditional approaches to meet students’ intrinsic needs and expectations (Cassidy, 2016). One of the expectations in both asynchronous, synchronous, and blended courses is the need for socialization and connection with peers and teachers (Ismailov and Laurier, 2021; Ismailov and Ono, 2021). Studies emphasize the importance of socialization and collaborative learning using various social networking tools, such as Twitter, Facebook or LMS-embedded chats (Wang et al., 2018). Accordingly, how UDL-based designs emotionally engage students in different gender and academic groups remains unclear.

### Cognitive Engagement in Universal Design for Learning-Based Online Courses

From a cognitive perspective, studies suggest that an effective online course using UDL principles typically features applicability and relevance of content, prompt and meaningful instructor feedback, and clear guidelines, course materials, and assignment parameters (Chiu and Mok, 2017; Rogers-Shaw et al., 2017; Chiu et al., 2020; Chiu and Lim, 2020). By exploring the influence of online UDL elements on students’ perception of learning and learning satisfaction, one study shows a significant correlation with integrated active learning activities, interactive engagement strategies, and robust assessment design and therefore recommends online instructors to widely utilize UDL principles to design their courses (Chen et al., 2018).

Studies also emphasize the effectiveness of high-quality and attractive videos to reach diverse student populations, especially those who want a high level of flexibility and more control over how they learn (Dinmore, 2019). While admitting that students in asynchronous online environments might still need teachers’ guidance, one study suggests that providing accessible review videos allowed students to practice and master content on their own, strengthening their independent study skills (Elliot et al., 2020). Although there is not enough empirical evidence regarding UDL’s comprehensive effect on learning outcomes (Capp, 2017), the existing research suggests that online courses based on UDL are cognitively more stimulating for diverse groups of students. Overall, the literature shows that although UDL is designed to foster diversity and inclusion, it may still have a different impact on different learners in diverse disciplinary contexts.

## Theoretical Framework

Self-determination theory (SDT) is a suitable psychological framework to assess how UDL-based teaching design caters to learner inclusion and diversity and to examine engagement in asynchronous online courses. By underscoring *“the basic human needs and the diversity of ways they are expressed and satisfied”* (Ryan and Deci, 2017, p. ix), the theory explicitly supports inclusive teaching practices. Specifically, the theory focuses on social-contextual factors that foster or hinder students’ thriving through the satisfaction of their basic psychological needs for autonomy, competence, and relatedness (Ryan and Deci, 2017). From SDT’s perspective, all students are inherently prone to learning, mastery, and connection with others, but these human tendencies are not spontaneous—they require nurturing conditions, such as need-supportive teaching behaviors, inclusive structures, and learning environments (Ryan and Deci, 2017, 2020; Han, 2021; Müller et al., 2021). When pedagogical designs effectively satisfy these needs, students are more likely to be motivated to engage in learning tasks (Hsu et al., 2019; Chiu, 2021a,b).

The autonomy need is described by SDT as a sense of voluntariness that is supported by experiences of interest and value (intrinsic motivation) but hindered by experiences of control, punishment and external reward (extrinsic motivation) (Ryan and Deci, 2017, 2020). A key factor that supports autonomy is the provision of choice through multiple learning modalities (Chiu, 2021c). The second need—competence—is a feeling of mastery and self-efficacy that are best satisfied within a well-structured pedagogical design that offers optimal challenges, positive feedback, and opportunities for growth (Ryan and Deci, 2017, 2020). A sense of competence diminishes in contexts in which challenges are too difficult, feedback is absent, or feelings of effectiveness are undermined by the perceived difficulty of learning tasks (Chiu et al., 2021; Ismailov and Ono, 2021). The third need from the SDT’s perspective concerns relatedness, enhanced by the sense of belonging and social connection. By feeling connected to others and by being a significant member of social groups, learners experience inclusion and belonging, for instance, by contributing to the group or learning with peers in formal and informal settings (Ryan and Deci, 2017; Chiu, 2021a).

By supporting autonomy, competence, and relatedness (SDT) as well as engagement, representation, expression/action (UDL), the two frameworks actively support diversity and curtail dominance in a classroom setting. Additionally, both SDT and UDL support person-centered approaches that maximize participant input and engagement in all learning situations and interventions (Ryan and Deci, 2017; Edyburn, 2020). Although SDT and UDL have many similar features that support inclusive pedagogy, surprisingly, to date, there are very few studies examining UDL from SDT’s perspective (Griful-Freixenet et al., 2020). Thus, it is necessary to further understand the effects of UDL on learners’ engagement and needs satisfaction through the prism of SDT.

## Materials and Methods

### This Study

Previous research found that UDL was effective in reducing barriers to learning, catering to classroom inclusion, and improving the learning process in students with diverse characteristics (Capp, 2017; Schreiber, 2017). However, most studies examined UDL mainly in MOOCs or in synchronous and blended environments. There has been a paucity of research on how fully asynchronous UDL-based courses cater to diversity and inclusion in a university setting. The SDT can explain how UDL engaged students in learning with technology from a needs satisfaction perspective (Ryan and Deci, 2020; Chiu, 2021a,b,c).

The goals of this study are to examine (i) whether pedagogically inclusive asynchronous pedagogic practices based on UDL could cater to inclusion and diversity across genders (female/male) and academic fields (Arts/Sciences); (ii) whether such instructions support learner engagement, performance, and needs satisfaction; and (iii) how the design supports learning. Accordingly, the following research questions were developed:

**RQ1.** Are there differences between male and female students in engagement, performance and needs satisfaction in UDL-based asynchronous courses?
**RQ2.** Are there differences between art and science students’ engagement, performance and needs satisfaction in UDL-based asynchronous courses?
**RQ3.** From a self-determination theory perspective, how meaningfully does UDL cater to inclusion and diversity in university-level asynchronous courses?

To achieve the research goals, this study designed and implemented two 15-week fully asynchronous online courses based on the UDL framework and tested their effectiveness among university students in a quasi-experimental setting.

### Participants

Participants were freshmen students (*N* = 225) attending English for academic purposes (EAP) online courses at a large university to the northeast of Tokyo, Japan. One group of students attended the “English reading skills” course (C1), and another attended the “English presentation skills” (C2). Both courses typically enroll very diverse and mixed populations from arts and science majors. These courses are also diverse in terms of gender composition. The researchers obtained ethical clearance from the university and consent from the participants.

### Learning Environment and Tasks

**FIGURE 2:**
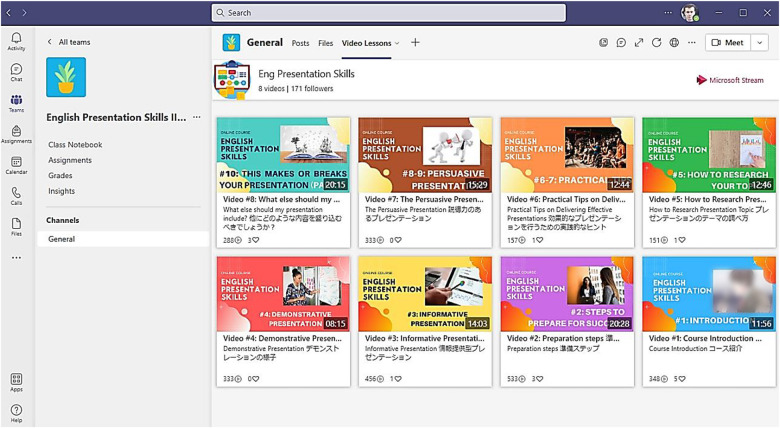
Microsoft Teams™—based course LMS. Microsoft and Microsoft Teams are trademarks of the Microsoft group of companies.

Both courses were taught to different groups of students for 15 weeks. All classes were redesigned to suit the online format during COVID-19. The university requested to conduct classes asynchronously to help freshmen adapt to university life and support those who experienced problems with online learning. The courses were based on the Microsoft Teams™ Learning management system (LMS). Prerecorded video lectures, instructional materials, and weekly learning tasks for both courses were designed in line with the UDL guidelines, as shown in Figure 2. Specifically, the following UDL principles and sub-criteria were integrated into the design of the two courses:

#### Engagement Principle

• Free choice of tasks by students for weekly topics.
• Asynchronous discussion boards.
• Age and ability appropriate tasks.
• Culturally/socially relevant and responsive assignments.
• Collaborative learning and reflection using chats.
• Frequent, timely, differentiated and specific teacher feedback on task.
• Varied degrees of freedom for acceptable performance, etc.

#### Representation Principle

• Use of multiple and flexible text, images, graphs, tables and other visual/auditory media in prerecorded lectures and learning tasks.
• Written bilingual transcripts for all videos and assignment guides.
• Closed captions in English and students’ language (Japanese).
• Use of visual organizers (KWL, concept maps).
• Activated prior knowledge through asynchronous discussion chats.
• Use of cues and prompts to draw attention to critical points on slides/tasks, etc.

#### Action and Expression Principle

• Course LMS compatible with assistive and screen-reader technology.
• Use of multiple interactive web tools (e.g., discussion chats, annotation tools, animated presentations).
• Lecture videos feature multiple and hands-on solutions to problems.
• Visible weekly online announcements on goals and schedules.
• Students advance access to assessment checklists and scoring rubrics.

### Design and Instruments

This quasi-experimental study adopted a sequential explanatory mixed method. In the first stage, objective statistical results were obtained through quantitative analysis. The focus was on understanding whether the UDL design was able to cater to diversity across gender and academic background based on needs satisfaction and engagement. To triangulate quantitative data, open-ended questionnaires were used to examine the students’ subjective responses to explain the findings of the quantitative data. Two experienced researchers independently conducted deductive content analysis to identify recurring themes in student responses (interrater reliability = 93%). This protocol helped synthesize and analyze students’ in-depth perspectives on how UDL design supported learners’ need satisfaction and engagement.

#### Needs Satisfaction

To assess students’ satisfaction of needs, researchers used a previously validated instrument (Standage et al., 2005). The instrument originally developed to assess needs satisfaction in physical education showed acceptable internal reliability for measuring students’ perceived autonomy (Cronbach’s α = 0.80), competence (α = 0.87), and relatedness (α = 0.87). To fit the study’s goals and learning context, all thirteen items were slightly modified. 7-scale Likert statements were used. Items related to perceived competence included three items, such as “I have some choice when choosing the topic and researching for my online presentation/reading tasks,” “I have a say regarding what skills I want to improve when making my online presentation/doing reading assignments,” and “I can decide which activities and tools I want to use when making my online presentation/doing reading assignments.” Items on perceived competence included five statements. Three example items were “I think I am pretty good at making online presentations/reading in English,” “I am satisfied with my ability to make online presentations,” and “I feel pretty confident about making online presentations/doing reading tasks in English.” Finally, items on perceived relatedness included five statements. Three example items were “With the other classmates in my online presentation class, I feel close,” “With the other classmates in my online presentation class, I feel valued,” and “I’d like a chance to interact with my classmates more often.”

#### Learner Engagement

Students’ behavioral engagement in asynchronous online courses was assessed using the instrument developed and validated by Skinner et al. (2009) with acceptable internal reliability (α = 0.72). The five items were slightly modified to fit this study’s goals. The three examples included “When I’m in my online presentation/reading class, I listen and read very carefully,” and “When I’m in my online presentation/reading class, I participate in asynchronous discussions.” Emotional engagement was measured with five items from the same study (Skinner et al., 2009) with acceptable reliability (α = 0.82). Likewise, the five items were slightly modified to fit this study’s goals. They included “When I prepare for my online presentation/reading tasks, I feel …” followed by “interested,” “important,” and “good,” “Making online presentation/reading in English is fun,” and “I enjoy learning new things when making an online presentation/reading in English.” To measure students’ cognitive engagement, four items from the study by Wang et al. (2016) were adapted. The items showed acceptable internal consistency (α ≥ 0.75) and were suitable for this study’s goals with slight modification. Two examples included “I work hard to prepare for my presentation/reading task, and I make sure that it is right,” and “I think about different ways to solve a problem when preparing or making my presentation/reading task.”

## Results

To answer RQ1 and RQ2, analyses of covariance (ANOVAs) were conducted to assess the differences between two groups in post-teaching mean scores. Descriptive statistics for all the variables were presented in Table 1. The variables were internally reliable, as all the α values ranged from 0.74 to 0.91 (where good > 0.70; Warner, 2013) and had sufficiently normal distributions (i.e., skewness less than 2.3, kurtosis less than 7.0, Byrne, 2010). Generally, behavioral, cognitive, and emotional engagement and perceived autonomy were above 5, while perceived competency and relatedness were approximately 4. All the variables met the assumption of homogeneity of variance, with Levene’s test returning *p* > 0.05 for ANOVAs.

**TABLE 1:** Descriptive statistics for all variables.

Group	Variable	Mean	*SD*
Male (*N* = 117)	Perceived autonomy	5.23	0.93
	Perceived competence	4.26	1.18
	Perceived relatedness	3.93	1.17
	Behavioral engagement	5.10	0.89
	Emotional engagement	5.38	1.08
	Cognitive engagement	5.48	0.88
Female (*N* = 108)	Perceived autonomy	5.21	0.93
	Perceived competence	4.11	1.20
	Perceived relatedness	4.02	0.96
	Behavioral engagement	5.06	0.86
	Emotional engagement	5.41	0.96
	Cognitive engagement	5.32	0.91
Science (*N* = 116)	Perceived autonomy	5.20	0.90
	Perceived competence	4.20	1.13
	Perceived relatedness	3.95	0.95
	Behavioral engagement	5.05	0.81
	Emotional engagement	5.38	0.94
	Cognitive engagement	5.41	0.88
Arts (*N* = 109)	Perceived autonomy	5.24	0.96
	Perceived competence	4.17	1.26
	Perceived relatedness	3.99	1.20
	Behavioral engagement	5.12	0.95
	Emotional engagement	5.40	1.10
	Cognitive engagement	5.40	0.92

To answer RQ1, ANOVAs showed that there were no significant differences between male and female students in perceived autonomy, [*F*(1, 224) = 0.02, *p* = 0.88, competency], [*F*(1, 224) = 0.90, *p* = 0.34], relatedness [*F*(1, 224) = 0.38, *p* = 0.54], and behavioral [*F*(1, 224) = 0.15, *p* = 0.70], emotional, [*F*(1, 224) = 0.05, *p* = 0.83] and cognitive, [*F*(1, 224) = 1.84, *p* = 0.18], engagement.

For RQ2, the analyses also revealed that there were no significant differences between science and art students in perceived autonomy, [*F*(1, 224) = 0.09, *p* = 0.77], competency, [*F*(1, 224) = 0.03, *p* = 0.85], and relatedness [*F*(1, 224) = 0.06, *p* = 0.81], and behavioral [*F*(1, 224) = 0.33, *p* = 0.57], emotional, [*F*(1, 224) = 0.01, *p* = 0.91] and cognitive, [*F*(1, 224) < 0.001, *p* = 1.00], engagement.

Overall, the analyses suggested that students of different genders and disciplinary backgrounds had the same level of needs satisfaction and engagement.

To answer RQ3 and triangulate quantitative data, the open-ended questionnaire responses were additionally analyzed using the SDT framework and the three dimensions of engagement.

• Autonomy: The data, as quoted verbatim, showed that all the students felt that they were given many freedoms to express creativity and knowledge and many task options to choose from, as shown in Table 2, points a, b, c.
• Competence: Most students also believed that the course media, tools, and contents were well balanced to cater to students’ diverse skills, preferences, and personal circumstances (points h, i, j).
• Relatedness: However, many students felt that despite engaging in course design, chat discussions and a few paired activities, the lack of face-to-face instruction and real-time communication inhibited students’ sense of relatedness and socialization (points d, f, g). The latter was more often reported by students taking C2 (“English presentation skills”). Many students taking C1 (“English reading skills”) felt the same, but they also noted that due to the course specifics and personal qualities (e.g., shyness), the lack of perceived relatedness did not hinder the learning process (see point e).

**TABLE 2:** Student responses related to needs satisfaction.

	Student responses	Needs
	a) I was happy to do assignments on my favorite topic, because it allowed me to explain in detail to everyone what I like (C1-S17). b) I felt that I had many options to study. I was able to learn English in a variety of ways, such as watching videos, reading handouts, making presentations and essays, talking with friends in chat, and getting feedback from my teacher (C2-S92). c) I think I had many options. Especially, for my presentations I was able to choose from many types and chose more easy one to feel more confident (C2-S64).	Autonomy
	d) You don’t know if you are studying well because you cannot see other classmates doing most of the English activities or assignments (C1-S44). e) I felt disconnected from others, because, I had limited chance to read together with classmates in real time. However, I prefer studying online alone, so I was still satisfied with this format (C1-S6). f) What we did this term was similar to what I’d done in high school, and I wasn’t too excited. I want to do something more active, like working with my classmates in the classroom to make a presentation (C2-S44). g) I had friends in the same group so I could discuss my presentation face-to-face, but if I did not, I think I’d feel totally disconnected (C2-S19).	Relatedness
	h) First, you can stop the video and understand contexts better. Second, you can watch lecture videos anytime. In addition, finally, you don’t need to move from room to room, so you can prepare for the class easier (C1-S53). i) I was very impressed with the presentation in the video lessons. It was very easy to understand. I was also impressed by how easy it was to use Microsoft teams and how easy it was to submit assignments (C1-S49). j) I was interested in this course because all tasks were so valuable. Especially, TED talk examples were nice. I could easily follow the course (C2-S90).	Competence

Overall, these results suggested that while UDL design could cater to diversity and inclusion in asynchronous courses, the learning outcomes were not linear due to differences in disciplinary environments as well as learners’ personal characteristics.

Regarding learner engagement, many students reported that the clarity and novelty of content, visual aids, length of videos, and user interface positively affected their behavioral engagement (see points k, l, m, Table 3). In addition, the lecturer’s speaking rate, dynamic animations used in prerecorded lectures, and integration of real-life demonstrations, such as TED talks, could help students be cognitively engaged (see points q, r, s). Finally, many students felt emotionally engaged with the course (points o, p). However, as with the lack of perceived relatedness, some students felt emotionally disengaged because of the absence of regular face-to-face interaction with classmates and instructors, as shown by point n in Table 3.

**TABLE 3:** Student responses related to engagement.

	Student responses	Engagement
	k) I also liked the fact that the videos were over in less than an hour, and the fact that I could check the assignments in the Teams section (C1-S6). l) I felt motivated to do the assignment because it was not too much and the content was interesting (C1-S13). m) The sound effects of the video content made it clear where to focus and where not to. In addition, I was able to deepen my understanding through images (C1-S88).	Behavioral
	n) One of the disadvantages I found in [C1] was that I could not see the teacher’s face in real, and I could not see the faces of my classmates who were also taking the class with me. This made me feel lonely, as if I was working on the class all by myself (C1-S63). o) When I took this course and do its activity, I felt good. When I finished making a presentation, I got satisfaction and felt important (C2-S16). p) I didn’t feel overwhelmed and stressed at all. I want to make a presentation again (C2-S33).	Emotional
	q) I could understand even thought I did not know some words because there were many images in the videos. Additionally, the speed of talking was good for me to understand (C1-S35). r) The videos of this class were the shortest in all classes which I take in this semester. The videos were more colorful and fuller of pictures than other lessons, so I could understand well (C1-S8). s) I carefully watched videos and read materials. In particular, TED talks were very interesting and attractive presentations, so I looked back over and over again (C2-S77).	Cognitive

## Discussion

This study designed and implemented two asynchronous UDL-based online courses and assessed their effectiveness among university freshmen in a quasi-experimental setting. The research team aimed to examine whether a universal instructional design was able to cater to inclusion and diversity across genders and academic characteristics and how the teaching design supported learners’ needs satisfaction and engagement. In this section, the paper reported four empirical implications and made several theoretical contributions and practical recommendations.

### Empirical Implications

First, both male and female students equally engaged in their learning in the asynchronous course and equally perceived the needs support from the course’s universal design. Similarly, there were no differences between science and art students in their needs satisfaction and engagement in course learning activities. These findings were aligned with SDT-based studies that suggested that needs satisfaction can stimulate student engagement in the course (Chiu, 2021a,b). From SDT’s perspective, all students, irrespective of their diversity, were intrinsically inclined toward learning and mastery, but these human tendencies necessitated fostering conditions, such as need-supportive teaching behaviors, inclusive design, and learning environments (Ryan and Deci, 2017, 2020). When teaching designs satisfied these needs, students were more likely to be motivated to engage in learning tasks (Hew and Cheung, 2014; Hsu et al., 2019; Chiu, 2021a). This study’s results also found strong support in UDL-based studies (Rao et al., 2015; Fidalgo and Thormann, 2017; Herrara Nieves et al., 2019).

**FIGURE 3:**
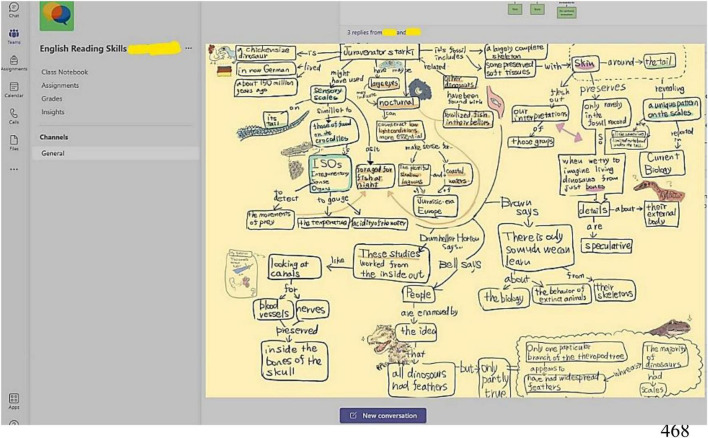
Students’ use of various tools, such as concept maps and KWL. Microsoft and Microsoft Teams are trademarks of the Microsoft group of companies.

Second, the qualitative data found that although universal designs could cater well to inclusion along with supporting autonomy and competence, they nonetheless did not fully satisfy learners’ needs for relatedness. For example, UDL-based asynchronous courses supported autonomy by providing the choice of multiple learning modalities (Chiu, 2021c). Similarly, such designs were effective in enhancing learners’ perceived competence and self-efficacy by offering optimal challenges, timely and positive feedback, and practically relevant assignments (Ryan and Deci, 2017; Chiu et al., 2021; Ismailov and Ono, 2021). For example, when students were given multiple options for completing certain tasks, they selected those types of assignments that matched their perceived competence and self-efficacy. Namely, when science-majoring students were completing concept maps, most of them tended to select themes related to their field, such as geology, engineering, etc. (see Figure 3). Despite these merits, an asynchronous format of learning with the universal design was not as effective as peer learning in real-time, the latter markedly increasing the available support for sustained engagement (Chiu and Hew, 2018; Wang et al., 2018). It is further explained in the next implication.

**FIGURE 4:**
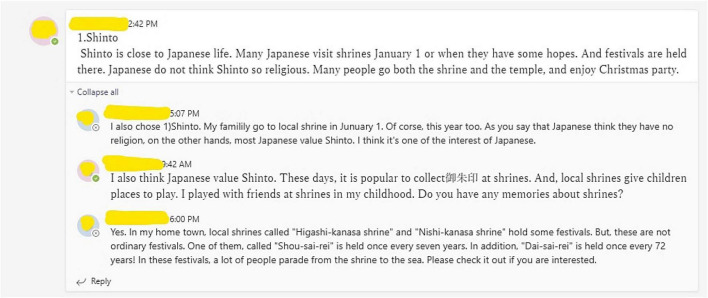
Asynchronous discussion board. Microsoft and Microsoft Teams are trademarks of the Microsoft group of companies.

Third, this study confirmed that relevant content, clear guidelines, course materials (Rogers-Shaw et al., 2017; Chen et al., 2018), and high-quality and attractive prerecorded videos (Dinmore, 2019; Elliot et al., 2020) enhance cognitive and behavioral engagement. However, this study also established that similar to the lack of perceived relatedness, emotional disengagement occurred in students who learned best when closely socializing with peers and teachers. Although the two UDL-based online courses used tools to promote learners’ self-expression and a sense of belonging to a community (see Figure 4), such as discussion boards, asynchronous chats, and 1-on-1 presentations (Herrara Nieves et al., 2019), they could not fully replace face-to-face or synchronous online interaction conducted through video conferencing software. Previous studies found that courses that were blended with live and interactive approaches satisfied students’ intrinsic needs and expectations (Cassidy, 2016; Ismailov and Ono, 2021). Additionally, by feeling closely connected with peers and teachers, learners experience better inclusion and belonging (Ryan and Deci, 2017; Chiu, 2021a; Ismailov and Laurier, 2021).

Finally, the present study found that the levels of social interaction and universality in such courses were also dependent on the learning goals of each subject (e.g., Reading vs. Speaking courses) and students’ personal characteristics and social circumstances (shy vs. outgoing; introverted vs. extraverted; had a part-time job vs. was flexible; had limited vs. unlimited access to Internet). For example, some students in this study first complained about the lack of regular live interactions with peers, but then they admitted that it would be “very stressful” (C2-S76) for them to participate because of their “own shyness” (C1-S32), “scheduling issues” (C1-S6), “poor Internet/broken PC” (C1-S92; C2-S49) or “some not-so responsible classmates” (C1-S50). Interestingly, Japanese female students across both Arts and Sciences tended to prefer slightly less socially interactive live sessions than males did (see Table 1), citing their “shyness” (C1-S32) or “embarrassing English” (C2-S84). In addition, many freshmen in this study mentioned that although the courses offered “many interesting task options” (C2-S22) to choose from, at times it was too hard for them to decide, they preferred teachers to provide only “fewer but very very good options” (C1-S17). The need for contextualization were discussed further as part of practical recommendations.

### Theoretical Implications

This paper made a theoretical contribution to understanding UDLs from the perspective of SDT. The idea that offering multiple options for classroom engagement, representation, and action and expression—the three core principles of UDL—made learning inclusive originated in cognitive neuroscience (Rose and Meyer, 2002; Burgstahler, 2021). Surprisingly, to date, only a few studies looked at UDL through the prism of social psychology, such as SDT. The results of the present study showed that many of the UDL guidelines are strongly supported by the SDT needs satisfaction framework.

First, the need for autonomy seen as voluntariness and initiative (Ryan and Deci, 2017) found a strong connection with UDL, which stressed that offering learners choices increased self-determination and pride in accomplishment and enhanced the feelings of belonging and ownership of activities (CAST, 2018). Thus, both SDT and UDL supported the premise that teachers should allow students to participate in the design of classroom activities and encourage learners to set their own personal academic and behavioral goals. Second, perceived competence seen by SDT as the feeling of mastery and self-efficacy (Ryan and Deci, 2017) equally supported the UDL framework. Inclusive design provided options for self-regulation and sustaining effort by heightening the relevance of goals and objectives, varying demands, and resources to optimize learning challenges, and increasing mastery-oriented feedback (CAST, 2018; Burgstahler, 2021).

Last, SDT’s focus on relatedness is strongly echoed by UDL, which helps learners feel comfortable communicating and collaborating within a community of learners (CAST, 2018). Indeed, this study demonstrated that by supporting autonomy, competence, and relatedness (SDT) as well as engagement, representation, expression/action (UDL), the two frameworks proactively nurtured diversity and inclusion and improved learner performance and satisfaction.

### Practical Recommendations

The study offers one practical suggestion for instructional designers and two recommendations for asynchronous online instructors in tertiary education teaching students with and without disabilities. The main suggestion for instructional designers is that the principles of UDL should not be approached as a “one-size-fits-all framework.” Indeed, UDL effectively caters to diversity by reducing physical, cognitive, intellectual, and organizational barriers (Rose and Meyer, 2002; Capp, 2017), but not all learners may necessarily see these conditions as “barriers,” as this study reveals. This study shows that “generalized UDL” may have a different impact on different learners in diverse disciplinary contexts. To optimize a UDL-based online course, designers and instructors should first carefully examine relevant factors, including external contexts that demand changes in the course, the course’s existing features, learner characteristics and needs, and the nature and requirements of the course content, activities, and assessments (Cai and Robinson, 2021; Jiang and Zhang, 2021). This can also be achieved through regular observation of teacher-student classroom interaction and provision of necessary support and adjustments when the course is still ongoing (Cotán et al., 2021). In short, UDL course designs should be approached as dynamic systems requiring constant monitoring and regular contextual adjustments.

For teachers, our first recommendation is to scrutinize the UDL guidelines themselves and adjust them to their specific courses without relying too much on institutionally recommended settings. The UDL has been in making for many years, and the current version (last accessed 19 November 2021)^1^ offers a wide variety of options for recruiting learner interest, sustaining effort and persistence, and self-regulation (CAST, 2018).

Our second recommendation is to optimize the level of social interaction in asynchronous courses. We believe that teachers should include face-to-face or, when impossible, synchronous online sessions and activities. Since students have different preferences in an asynchronous university setting (i.e., some want to study alone, while others want to study in groups), teachers may conduct “preference polls” at the beginning of each course and divide the class into small cohorts based on their modal preferences. Alternatively, teachers may explicitly instruct students that any format of collaborative learning is acceptable if baseline rules are followed by everyone. Indeed, the last point should be tried with caution, as it requires a collective effort, constant teacher scaffolding, and especially with freshmen groups, more direct task instruction (Ismailov, 2021a,b,c).

## Conclusion

Several conclusions were drawn from this study. First, all students (male/female, arts/science majors) equally engaged in their learning in the asynchronous course and equally perceived the needs support from the UDL (RQs 1-2). All students, irrespective of their differences were intrinsically inclined toward learning and engagement, but as our study confirmed inclusive education necessitated additional fostering conditions, such as need-supportive teaching, engaging instructional design, and a real-time learning environment. This was supported by our second conclusion in that despite its many benefits an asynchronous format of learning based on UDL nonetheless was not as effective as peer learning in a synchronous or physical classroom environment. Namely, this study showed that although universal design effectively supported autonomy and competence needs, it did not fully satisfy students’ needs for relatedness (RQ 3). Echoing previous studies, it was found that courses that were blended with live and interactive approaches might better satisfy students’ intrinsic needs and expectations. By learning with peers and teachers in real-time, students tended to perceive more inclusion, social connection, and belonging.

## Limitations and Future Directions

First, to assess whether UDL-based courses catered for inclusion and diversity in an asynchronous environment from the perspective of self-determination, this study used “gender” and “academic background” as the two primary measures. More studies are needed to extend our methodology and to understand how other learner characteristics, such as disability, age, ethnicity, and learning styles, influence the effectiveness of teaching based on universal design. Secondly, in the experiments, this study used a within-subjects design to test the effectiveness of UDL-based online courses without a control group. For stronger internal validity, future studies could include a control group, if possible. Without control groups, it might be harder to be certain that some outcomes were caused by the experimental conditions and not by other variables. Finally, as many universities worldwide were found actively internationalizing their programs by offering content courses in certain lingua franca, such as through English Medium Instruction (Ismailov et al., 2021), future studies could use “prior academic background” and “multilingualism” of exchange students as additional variables to measure classroom inclusion and diversity. In sum, since this study focused only on EAP courses with students using their second language (L2), future research on asynchronous online education with universal design should address various disciplinary contexts in Arts and Sciences in both first and second language settings.

## Data Availability Statement

The raw data supporting the conclusions of this article will be made available by the authors, without undue reservation.

## Ethics Statement

The studies involving human participants were reviewed and approved by the Ethics Committee, Faculty of Humanities and Social Sciences, University of Tsukuba. The participants provided their written informed consent to participate in this study.

## Author Contributions

Both authors listed have made a substantial, direct, and intellectual contribution to the work, and approved it for publication.

## Conflict of Interest

The authors declare that the research was conducted in the absence of any commercial or financial relationships that could be construed as a potential conflict of interest.

## Publisher’s Note

All claims expressed in this article are solely those of the authors and do not necessarily represent those of their affiliated organizations, or those of the publisher, the editors and the reviewers. Any product that may be evaluated in this article, or claim that may be made by its manufacturer, is not guaranteed or endorsed by the publisher.

## Footnotes

1
https://udlguidelines.cast.org/
